# Supplementary material for: The Role of Parental Adherence to the Mediterranean Diet and Family Time Together in Children’s Weight Status: The BeE-School Project
Source: Nutrients. 2024 Mar 22;16(7):916. doi: 10.3390/nu16070916 (PMC11013135; doi:10.3390/nu16070916)
Supplement: Supplementary file 1 [file nutrients-16-00916-s001.zip › nutrients-2905587-supplementary.pdf]

Supplemmentary table

Table S1. Binary logistic regression analysis of the associations between children's weight status with the interaction MEDAS*highest parents education level.

	**Children’s weight status**
Interaction MEDAS*highest parents education level	**0.328(0.121; 0.892)+**

Note: values expressed as OR (CI 95%). Variables in the model: MEDAS (categorical) and highest parents education level (categorical). +**p-value=0.029**
